# Cone-beam computerized tomography study of the temporomandibular joint with different vertical bone facial types in adult females with class II bone

**DOI:** 10.1097/MD.0000000000042214

**Published:** 2025-05-02

**Authors:** Ke Zhang, Guannan Wang, Haojie Hu, Zhiqian Li, Xin Li

**Affiliations:** a Affiliated Stomatological Hospital of Jinzhou Medical University, Jinzhou, Liaoning; b School of Stomatology, Shenyang Medical College, Shenyang, Liaoning; c Luohe Central Hospital, Luohe, Henan; d Key Laboratory of Human Ethnic Specificity and Critical Illness in Liaoning Province (LPKL-PHESCI), Shenyang, Liaoning; e Shenyang Key Laboratory for Phenomics, Shenyang, Liaoning; f Shenyang Key Laboratory of Prevention and Treatment of Systemic Important Diseases Associated with Oral Diseases, Shenyang, Liaoning.

**Keywords:** bony class II, cone-beam computerized tomography, temporomandibular joint

## Abstract

The aim was to study the morphological and positional characteristics of temporomandibular joints (TMJ) in adult females with different vertical bony facial types of bony class II. A total of 60 adult females with bony class II were divided into high-angle group, low-angle group, and an average-angle group in the Frankfort horizontal plane-gonion-gnathion angle (MP-FH). In the control group, there were 20 patients with bony class I homogeneous angle, and the ages of each group were between 18 and 35 years old. Dolphin software was used to generate lateral cranial views and perform fixed-point tracing. Invivo 5.3 software was used to reconstruct the 3-dimensional image of the TMJ, and the following items were measured under the view of the TMJ: (1) bony structures such as condyle and fossa and (2) condyle position. The statistical software SPSS27.0 was used to collate and analyze the data. There was no significant difference in bilateral TMJ measurements between the groups (*P* > .05). Compared with the other groups, the long axis of the condyle, short axis, and supra-articular space (SS) were larger, the fossa depth was deeper, articular eminence inclination was steeper, the long-term axis and the short axis of the high angle were smaller, the fossa depth, and articular eminence inclination were smaller, the anterior articular space was larger (*P* < .05), and the supra-articular and posterior spaces were smaller. The position of the condyle was mainly in the median and anterior positions in the control group, the anterior and posterior positions in the average angle group, the median and posterior positions in the low-angle group, and the posterior position in the high-angle group. The morphology and position of bilateral TMJ were basically symmetrical between the skeletal class II adult females and the control group, and the position and morphology of the TMJ in the vertical skeletal type of adult females with bony class II were different.

## 1. Introduction

The temporomandibular joint (TMJ) is the most important and complex joint in the oral and maxillofacial area, mainly composed of the condyle, temporal bone articular surface, articular disc, joint capsule, and articular ligaments, and is the only joint in the body that can rotate and slide at the same time.^[1]^ Normal TMJ morphology and structure not only maintain the function and structure of the oromaxillofacial system in conjunction with the occlusal of the dentition, masticatory muscles, and craniomaxillofacial morphology but also contribute to the stability of orthodontic therapy.^[2]^ There is a certain correlation between the morphological structure of TMJ and the stress it is subjected to, and occlusal factors may lead to changes in the stress acting on TMJ,^[3]^ so different types of malocclusion deformities also have an impact on the morphological structure of TMJ.

Bony class II malocclusion is a common malocclusion in clinical practice, and such patients often have severe bony imbalance, usually manifested by mandibular recession, protruding upper teeth, open lips and teeth, and no occlusal contact between anterior teeth.^[4]^ Patients with severe bony class II malocclusion are often unable to use anterior occlusions, which can lead to an increased burden on TMJ and an increase in the development of temporomandibular disorder (TMD). Therefore, it is necessary to explore and understand the TMJ characteristics of patients with bony class II malocclusion before orthodontic treatment.

Reviewing previous literature, although most scholars have studied the relationship between TMJ morphology, condyle position, and various malocclusion deformities and facial growth patterns, the conclusions have not been consistent,^[5,6]^ and there are few reports on the correlation between TMJ morphology, condyle position, and craniomaxillofacial morphology of adult female patients with type II bone. Therefore, cone-beam computerized tomography (CBCT, LargeV, Beijing, China), Dolphin Imaging 11.95 software and Invivo 5.3 software (Dolphin Imaging & Management Solutions, Chatsworth) were used in this project to measure and analyze the craniomaxillofacial morphology-related indexes and TMJ-related structures of adult to evaluate the differences in TMJ morphology and condyle position among adult female patients with different bone facial types and patients with osteopathic class I homoganges, and to explore the correlation between TMJ morphology and condyle position and craniofacial morphology and structure in adult female patients with osteopathic class II, so as to provide a reference for clinical diagnosis and treatment of these patients and make reasonable diagnosis and treatment plans.

## 2. Materials and methods

### 2.1. Subjects of the study

Some studies have shown that condyle morphology and location are somewhat influenced by gender.^[7]^ Therefore, to exclude the influence of gender factors on the study results, this study restricted gender and only selected women as the study object. Efficacy analysis was performed using the GPower software to determine the sample size. At test level α = 0.05, test efficacy 1-β  = 0.8, at least 76 participants should be included, on average of at least 19 participants per group.

Patients who had been admitted to the Department of Stomatology of Luohe Central Hospital from October 2018 to December 2022 and had CBCT images were selected, and 60 experimental groups were selected according to the inclusion and exclusion criteria, including 20 cases in the low-angle group, high-angle group and 20 cases in the average angle group, and 20 patients with bony class I average angle were selected as the control group. This study was reviewed by the Ethics Committee of Luohe Central Hospital (Batch number: Luoyi Ethics No. 2024-38). All of the patients gave their informed consent.

### 2.2. Inclusion criteria

Following subjects were included: (1) females aged 18 to 35 years; (2) the face is basically symmetrical; (3) good physical health, no rheumatism, rheumatoid and other systemic diseases; and (4) no missing teeth except the third molar.

Inclusion criteria for the control group are as follows:

(1) 0°＜subspinale-nasion-supramental angle (ANB)＜4.7°, homogeneous bone surface type: 22° ≤ MP-FH ≤ 32°;

(2) the occlusion is basically good (individual normal occlusion).

Inclusion criteria in the experimental group are as follows: sagittal bone surface ANB ≥ 4.7°; vertical grouping: according to Professor Fu Minkui’s vertical bone type classification method,^[8]^ high-angle bone type: MP-FH > 32°; low-angle bone surface type: MP-FH < 22°; and homogeneous bone surface type: 22° ≤ MP-FH ≤ 32°.

### 2.3. Exclusion criteria for all cases

Exclusion criteria are as follows:

(1) History of TMD, oral and maxillary trauma, orthodontic treatment, orthognathic treatment, and other systemic medical history;

(2) Pathological occlusal factors such as closed deep occlusion, locking occlusion, and crossbite;

(3) The lower jaw is significantly deviated;

(4) There is obvious abrasion of the occlusal surface of the tooth (the morphology of the occlusal surface changes and the height of the crown decreases);

(5) Poor quality of CBCT images, which affects identification and measurement.

### 2.4. CBCT data acquisition

The patient remains naturally upright and has his mouth closed, and the upper and lower dentition are intertwined. With the help of the head fixture, make sure that the median sagittal plane of the subject is perpendicular to the ground plane, so that the plane through the orbital ear and the tip of the nose is parallel to the ground plane, and at the same time align the center line of the cursor positioning system with the TMJ to complete the imaging of the TMJ area on both sides and save it in DICOM format.

### 2.5. CBCT image processing

#### 2.5.1. Acquisition and measurement of lateral cranial views

**Figure 1.**
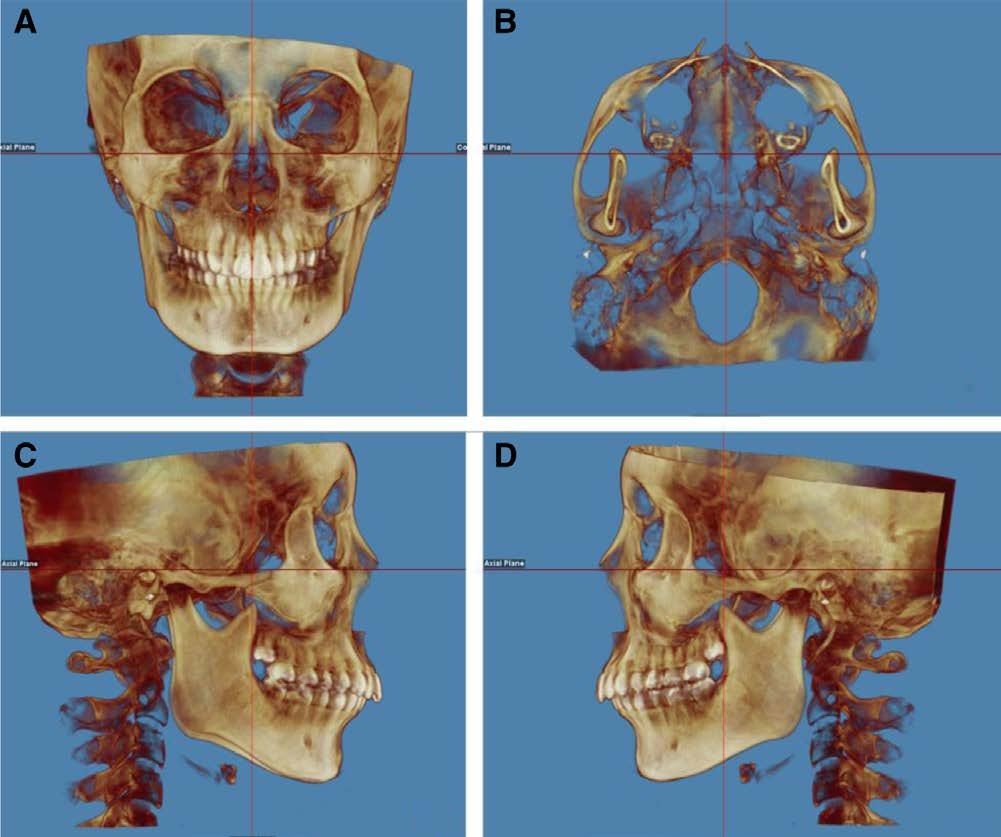
Correction of cephalic position.

**Figure 2.**
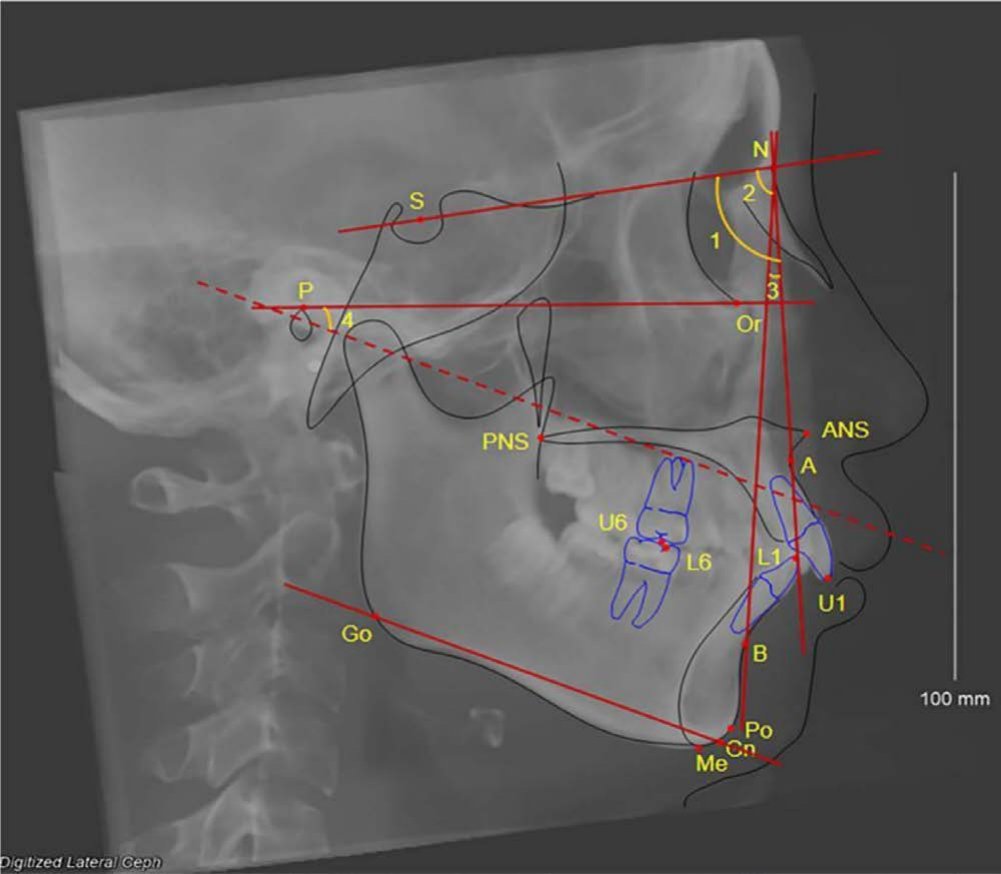
Cephalometric markers and measurement item.

To ensure the accuracy of the measurement data, the software should be used to calibrate the CBCT images of all patients with a unified standard, so that the bilateral orbital auricular planes are parallel to the horizontal plane (as shown in Fig. 1A, C, D), and the line between the nasal septum and the foramen magnum is parallel to the median sagittal plane (Fig. 1B). In the lateral view mode of the skull, the software was used to automatically generate the lateral view of the cranium, the cephalogram was measured, and the angle MP-FH formed by the SNA, sella-nasion-supramental angle (SNB), ANB, and the mandibular plane (Go-Gn) and orbital auricular plane was measured (Fig. 2).

### 2.6. Acquisition and measurement of temporomandibular joint images

#### 2.6.1. Three-dimensional reconstruction and measurement of the temporomandibular joint

**Figure 3.**
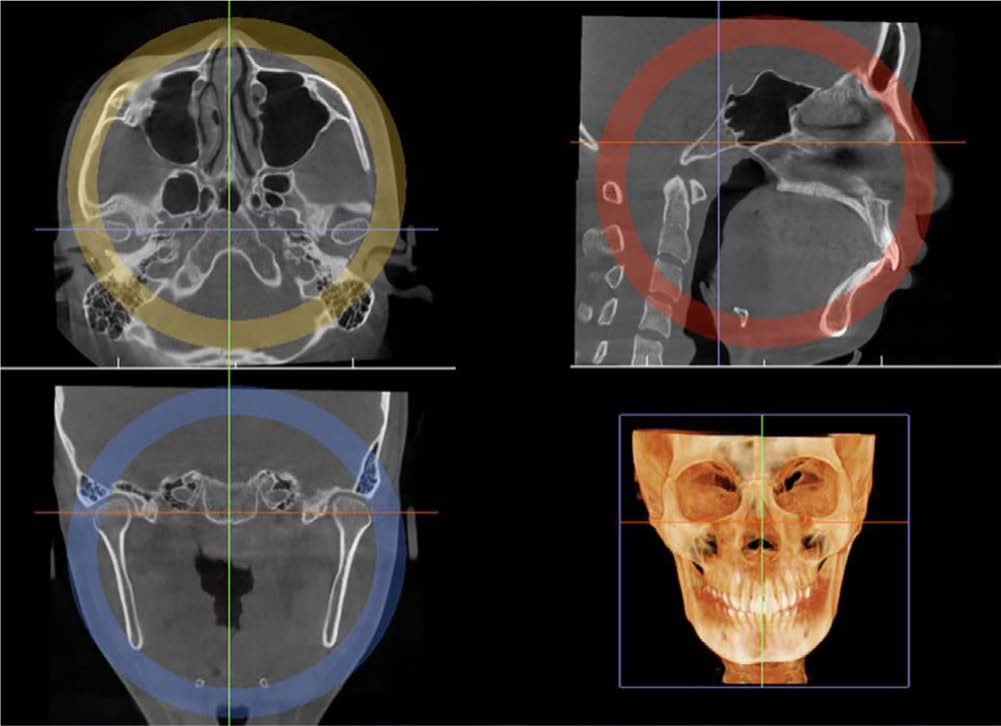
3D reconstruction view of axis, sagittal position, coronal position, and skull.

The DICOM format data were imported into Invivo 5.3 software for 3D reconstruction and correction so that the sagittal line of the axial position passed through the center point of the nasal tip, nasal septum, and foramen magnum. Make sure that the orbital luric plane is parallel to the horizontal plane, and that the left and right condyles are in the axial and coronal positions Basic symmetry (Fig. 3).

**Figure 4.**
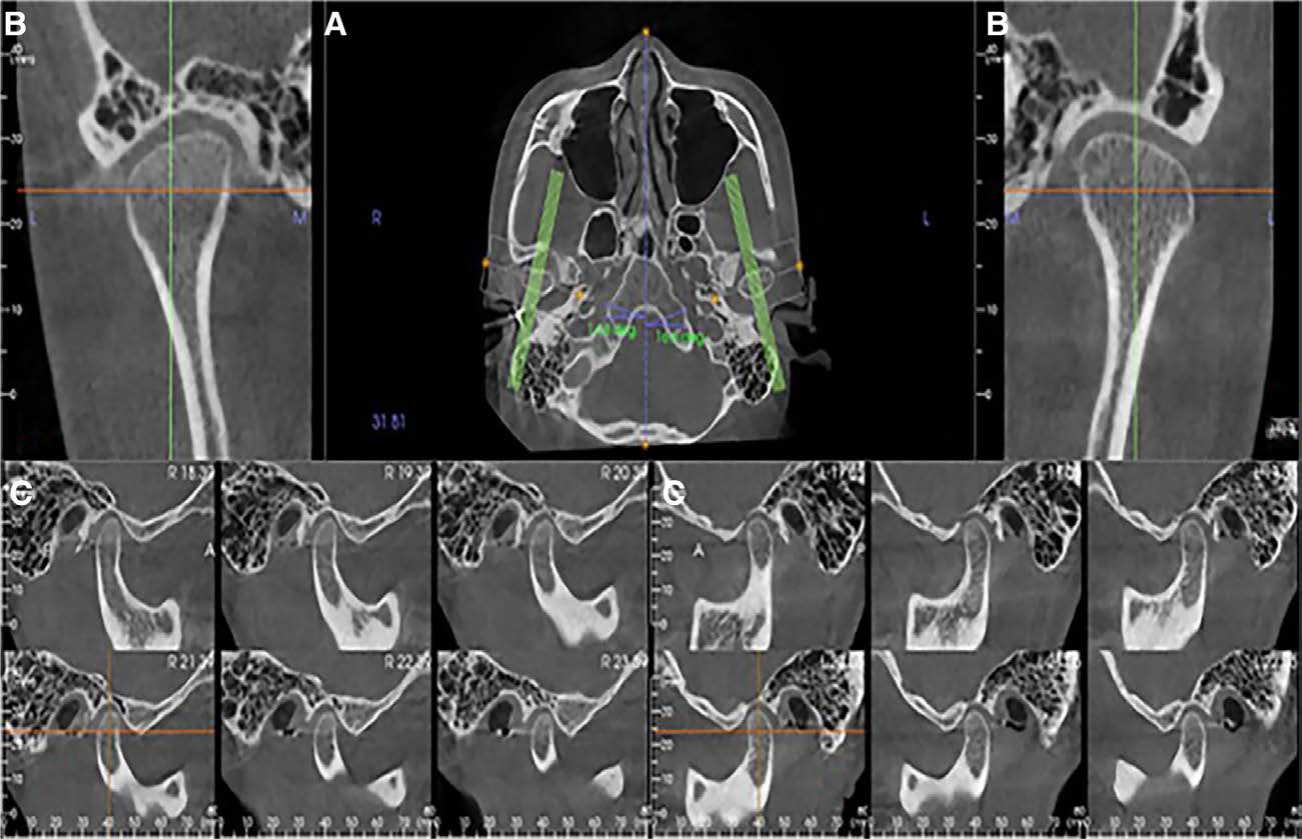
Corrected axial, coronal, and sagittal positions.

**Figure 5.**
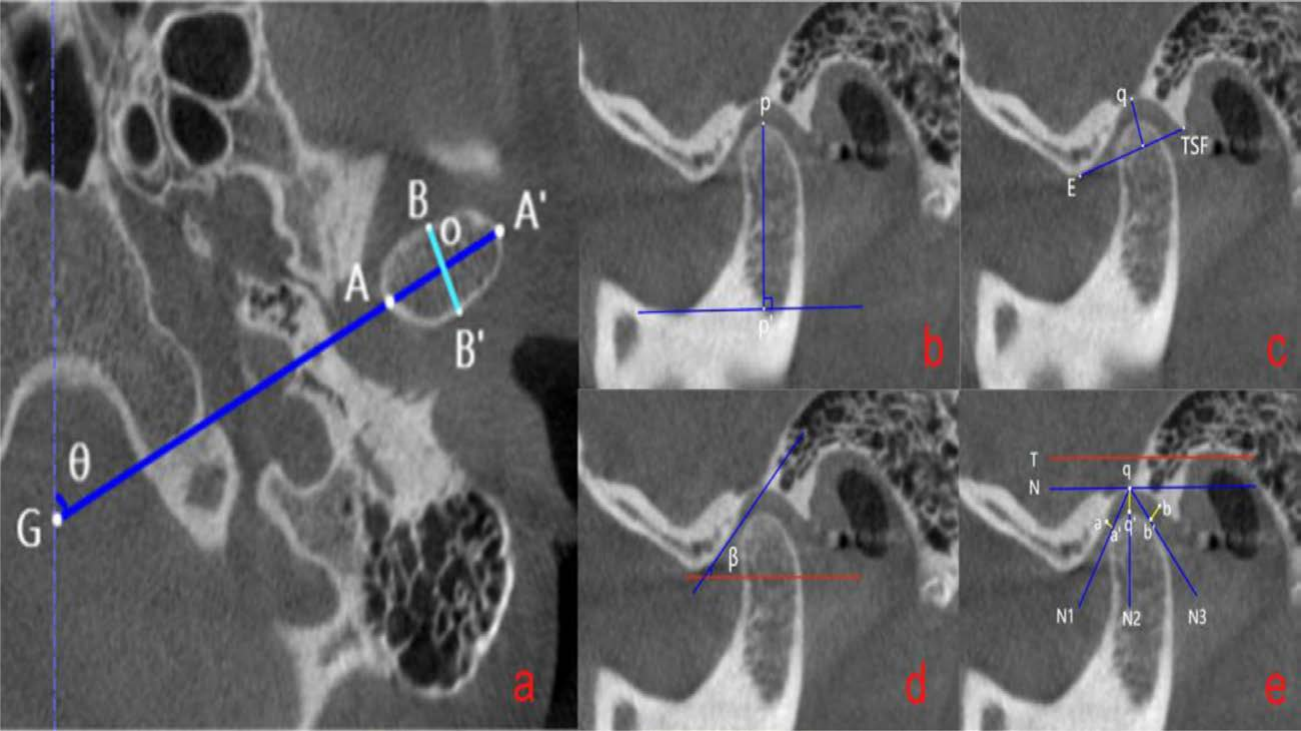
Corrected axial position measurement and corrected sagittal measurement. a: AA’: condylar long axis (LAC), BB’: condylar short axis (SAC), θ: condylar long-axis angle (CHA). a–d in order: pp’: condylar height (CH), qe: fossa depth (FD). E-TSF: socket width (WF), β: articular eminence inclination (AEI), aa’: prearticular space (AS), qq’: supra-articular space (SS), bb’: posterior articular space (PS). E-TSF=The lowest point of the joint nodule and the bulge and fissure.

Measurement items: The corrected axis position includes the long axis of the condyle (LAC), the short axis (SAC), and the long-axis angle (CHA) (Fig. 4). The corrected sagittal view includes condylar height (CH), fossa width (WF), fossa depth (FD), articular eminence inclination (AEI), anterior articular space (AS), supra-articular space (SS), and posterior space (PS) (Fig. 5).

The condylar position was assessed by the formula^[9]^ ln (PS/AS), which was characterized by anterior condylar displacement at ln (PS/AS) greater than 0.25 and posterior condylar displacement at ln (PS/AS) less than −0.25. When the ln (PS/AS) is between −0.25 and +0.25, the condyle is basically located in the middle of the fossa.

### 2.7. Statistical analysis

SPSS27.0 statistical software (IBM Corporation, Armonk) was used for analysis. The measurement data were expressed as x ± s, and the counting data were expressed as (n/%). All data were tested for normality. A paired *t* test was used for TMJ measurement items on the left and right sides of each group. One-way analysis of variance was applied for comparison of TMJ measurement items between different groups. For items with statistically significant differences, the least significant difference *t* test was used for pairwise comparison. The average value of the measurement data of the left and right joints in patients with different vertical bone surface types was calculated. Under the condition of normal distribution, the Pearson correlation test was used to analyze the correlation between the ANB angle, mandibular plane angle, and the average value of the measurement parameters of bilateral TMJ in patients with bony class II malocclusion.

## 3. Results

### 3.1. Comparison of measurement items on the left and right sides of TMJ in each group

There was no significant difference between the left and right measurement items in the 4 groups (*P >* .05), so the data from the 2 sides were combined into 1 group and then compared between groups (Table 1).

**Table 1:** Comparison of temporomandibular joints measurement items on the left and right in different groups (right-left), x̄ ± s, n = 20).

Measurement items	Control group	Low-angle group	High-angle group	Uniform-angle group
	Left and right side differences	Left and right side differences	Left and right side differences	Left and right side differences
LAC	−0.15 ± 0.36	−0.05 ± 0.90	0.19 ± 0.63	−0.29 ± 0.82
SAC	0.08 ± 0.48	0.09 ± 0.60	0.03 ± 0.38	0.12 ± 0.63
CHA	0.02 ± 3.89	0.26 ± 3.98	−0.41 ± 3.65	0.12 ± 3.13
CH	−0.19 ± 1.19	−0.15 ± 1.11	−0.04 ± 0.83	−0.10 ± 1.26
WF	−0.09 ± 0.82	−0.00 ± 0.81	−0.14 ± 0.78	0.07 ± 0.77
FD	−0.04 ± 0.51	−0.03 ± 0.36	0.13 ± 0.68	0.08 ± 0.56
AEI	1.13 ± 2.43	−1.74 ± 3.96	0.28 ± 2.70	0.65 ± 3.50
AS	−0.04 ± 0.12	0.04 ± 0.12	−0.03 ± 0.09	0.00 ± 0.10
SS	0.02 ± 0.14	0.14 ± 0.33	−0.05 ± 0.21	0.02 ± 0.22
PS	−0.01 ± 0.15	−0.03 ± 0.23	0.04 ± 0.10	−0.02 ± 0.14

### 3.2. Comparison of TMJ measurement items between different groups

#### 3.2.1. Comparison of TMJ measurement items between different vertical bone surface types of bony class II

Compared with the high-angle group, LAC, SAC, CHA, FD, and AEI in the low-angle group were higher than those in the high-angle group, and there was no statistically significant difference in WF (*P* > .05), while the AS in the low-angle group was smaller, SS and PS were relatively larger, and there was no statistically significant difference in other measurement values (*P* > .05) (Tables 2 and 3). LAC, FD, and AS in low-angle group were higher than those in the average-angle group, but there was no significant difference in other measurements (*P* > .05). When the high-angle group was compared with the average angle group, there was a significant difference only in AS, and the high-angle group was greater than the average angle group (*P* < .05).

**Table 2:** Comparison of each item of temporomandibular joints between different groups (mm/°, x̄ ± s).

Measurement items	Control group	Low-angle group	High-angle group	Uniform-angle group	*F*	*P*
LAC	18.24 ± 0.70	19.20 ± 1.07	17.48 ± 0.92	17.86 ± 0.96	25.71	＜.001**
SAC	7.61 ± 0.58	7.74 ± 0.59	7.40 ± 0.43	7.50 ± 0.60	2.74	.045*
CHA	70.44 ± 4.54	70.16 ± 4.30	67.03 ± 3.37	68.77 ± 3.91	5.94	＜.001**
CH	20.24 ± 1.29	20.62 ± 1.29	20.29 ± 1.27	20.66 ± 1.69	0.99	.399
WF	17.94 ± 1.06	17.63 ± 0.85	18.08 ± 1.18	17.93 ± 1.05	1.33	.267
FD	7.51 ± 0.65	7.98 ± 0.78	6.99 ± 0.70	7.22 ± 0.56	15.61	＜.001**
AEI	48.21 ± 2.58	49.49 ± 3.86	46.61 ± 3.24	48.09 ± 3.80	4.79	.003**
AS	1.89 ± 0.19	2.04 ± 0.22	2.22 ± 0.20	1.93 ± 0.26	18.23	＜.001**
SS	2.95 ± 0.33	3.09 ± 0.37	2.82 ± 0.25	2.96 ± 0.28	5.05	.002**
PS	1.99 ± 0.18	2.00 ± 0.22	1.88 ± 0.20	1.94 ± 0.21	2.85	.039*

* *P* < .05.

** *P* < .01.

**Table 3:** LSD *t* test results of temporomandibular joints measurements between experimental groups (x̄ ± s, n = 40).

Measurement items	Low-high	*P*	Low-average	*P*	High-average	*P*
	Mean difference		Mean difference		Mean difference	
LAC	1.72	＜.001**	1.34	＜.001**	−0.38	.067
SAC	0.34	.007**	0.24	.052	−0.09	.451
CHA	3.14	＜.001**	1.39	.127	−1.75	.056
CH	0.33	.286	−0.04	.898	−0.37	.232
WF	−0.45	.054	−0.29	.209	0.16	.497
FD	0.98	＜.001**	0.75	＜.001**	−0.23	.135
AEI	2.88	＜.001**	1.40	.067	−1.48	.054
AS	−0.18	＜.001**	0.11	.024*	0.29	＜.001**
SS	0.27	＜.001**	0.14	.053	−0.13	.055
PS	0.12	.009**	0.05	.233	−0.07	.151

* *P* < .05.

** *P* < .01.

#### 3.2.2. Comparison of TMJ measurement items between different vertical bone surface types of bony class II and control groups

Compared with the control group, the low-angle group with LAC was larger than the control group, the FD in the low-angle group was deeper, and the AS and SS in the low-angle group were larger, while there was no significant difference in PS (*P* > .05) (Table 4). Compared with the control group, the LAC, CHA, FD, and AEI groups were smaller than the control group, the AS of the high-angle group was greater than that of the control group, and the difference was statistically significant(*P* < .05). There was no significant difference between the average angle group and the control group (*P* > .05).

**Table 4:** LSD *t* test results for each measurement item of the temporomandibular joints between each experimental group and the control group (x̄ ± s, n = 40).

Measurement items	Low-control	*P*	High-control	*P*	Uniform-control	*P*
	Mean difference		Mean difference		Mean difference	
LAC	0.96	＜.001**	−0.76	＜.001**	−0.38	.068
SAC	0.13	.288	−0.20	.101	−0.11	.373
CHA	−0.27	.763	−3.41	＜.001**	−1.67	.068
CH	0.38	.223	0.05	.880	0.42	.179
WF	−0.31	.187	0.14	.538	−0.02	.949
FD	0.47	.002**	−0.51	＜.001**	−0.28	.062
AEI	1.29	.094	−1.60	.038*	−0.12	.876
AS	0.14	.004**	0.33	＜.001**	0.03	.526
SS	0.14	.042*	−0.13	.068	0.01	.920
PS	0.01	.792	−0.11	.019*	−0.04	.353

* *P* < .05.

** *P* < .01.

### 3.3. Distribution of the position of the condyles in the fossa in different groups

In the control group, the condyle was predominantly median. Among the different vertical bony surface types of bony class II, the condyles in the low-angle group were mainly in the median and posterior positions. The condyles in the high-angle group were dominated by the median and posterior positions. The condyles in the average angle group were mainly in the anterior and posterior positions (Table 5).

**Table 5:** Distribution of condyle position in each group.

	Control group (%)	Low-angle group (%)	High-angle group (%)	Uniform-angle group (%)
Anterior	10 (25.00)	8 (20.00)	4 (10.00)	15 (37.50)
Median	24 (60.00)	20 (50.00)	15 (37.50)	11 (27.50)
Posterior	6 (15.00)	12 (30.00)	21 (52.50)	14 (35.00)

### 3.4. Correlation analysis of bony class II TMJ measurement items with cephalometric items

To compare the relationship between TMJ measurement items of class II patients and ANB angle and MP-FH of cephalometric measurement items, TMJ measurement values of low-angle, high-angle, and average-angle groups of adult female class II patients with bone were combined into class II patients, and the average value of bilateral TMJ measurement items of each patient was calculated as the measurement value of the patient item. Pearson correlation test was used for correlation analysis, and the results showed that ANB angle was negatively correlated with CHA and positively correlated with AS. MP-FH was significantly negatively correlated with LAC, FD, AEI, SS, and PS, while MP-FH was significantly positively correlated with AS (Table 6).

**Table 6:** Correlation analysis between osseous class II temporomandibular joints measurement items and cephalometric indexes (n = 60).

Measurement items	ANB	*P*	MP-FH	*P*
	*r*		*r*	
LAC	−0.179	.172	−0.510	＜.001**
SAC	−0.126	.336	−0.190	.147
CHA	−0.276	.033*	−0.219	.093
CH	0.015	.908	−0.061	.646
WF	0.163	.214	0.218	.094
FD	−0.111	.397	−0.471	＜.001**
AEI	−0.226	.083	−0.343	.007**
AS	0.268	.038*	0.343	.007**
SS	0.008	.953	−0.319	.013*
PS	−0.242	.062	−0.304	.018*

* *P* < .05.

** *P* < .01.

## 4. Discussion

The only movable joint of the oral and maxillofacial area is TMJ, which is located in the deep craniomaxillofacial area and has a complex anatomical structure, and the morphology and structure of TMJ are closely related to orthodontic treatment, but due to its special location and complex anatomical structure, the internal tissue structure changes of the TMJ region cannot be accurately understood only through clinical examination, and imaging examination is usually needed as an auxiliary means to reflect the changes in the TMJ area. CBCT is considered the preferred modality for assessing the bony structure of the TMJ because it can visualize TMJ anatomy and lesions from different sections and multiple views.^[10]^ Since this project mainly studied the bony structure of TMJ and did not involve soft tissue structures such as joint discs, this study selected CBCT to conduct 360° digital 3D scanning of the research subjects, and combined with Invivo 5.3 measurement software for processing, and analyzed the differences of TMJ between patients with different vertical bone surface types by measuring the TMJ morphological structure of adult women with bone class II at different levels.

TMJ is the only biarticular structure in the oral and maxillofacial area, which can perform both sliding and rotation, as well as asynchronous movements, so the structural and morphological symmetry of TMJ is essential for maintaining good facial function. In this study, the measurement items of left and right TMJ in adult women were compared, and the results showed that although the measured values of left and right TMJ were different in each group, the difference was not statistically significant, indicating that the morphological structure and condyle position of TMJ in patients with different sagittal and vertical bony types were basically symmetrical. In the past, some scholars^[11]^ evaluated the CBCT images of the TMJ of patients with different malocclusions and found that there was no significant difference in the morphology of the left and right TMJs. Yun et al^[7]^ found that there were no statistically significant differences between the left and right TMJs in terms of condylar size, condylar horizontal angle, FD, and joint space. The results of Chae et al^[12]^ showed that there were significant differences in the position of the left and right condyles, with the left side of the SS, the PS, and the slope of the joint tubercle being greater on the left than on the right, which differed from our results, which may be related to the different selection criteria of the study subjects. In this study, factors that may affect the symmetry of TMJ, such as obvious tooth wear, dentition loss, and jaw deviation, were excluded from this study, so as to avoid abnormal joint morphology interfering with the research results. On the other hand, adult females between the ages of 18 and 35 were selected for the study in this project, which avoided the interference of age and gender in the research results, so the research results obtained were highly reliable.

In terms of condylar morphology, the anterior-posterior diameter and inner and outer diameter of the condyle were the smallest in the high-angle group and the largest in the low-angle group, and the correlation analysis showed that the angle of the mandibular plane was negatively correlated with the LAC. It is speculated that this may be due to the weak masticatory muscles, more elongation of mandibular molars, easy formation of open occlusion or opposite blades of anterior teeth, and small inclination and condylar inclination, resulting in insufficient high development behind the mandible, resulting in limited condylar growth and development, so that the condylar process growth is small, and the long and short axes are small. In contrast to patients with high angles, patients with low angles have strong masticatory muscles that stimulate the growth and reconstruction of the condyle, with large long and short axes. The control group was mainly a class I homogeneous bone-faced type, which had a high proportion of coordination between the front and back, so the development of the condyle was basically normal. Previous studies have also shown that patients with low angles have thicker condyles, while patients with high angles have relatively small condyles.^[13,14]^ The connection between the small condyle and the socket is relatively loose, resulting in poor functional coordination and increasing the risk of displacement. The large condyle is better matched to the socket, and the joint is relatively more stable. Therefore, it is necessary to pay close attention to the changes in the position of the condyle in patients with high angles. In this study, there was no significant difference in the measured value of CH between the groups, which is consistent with the view of most previous scholars that there was no difference in CH in patients with different vertical bony facial types of bony class II.^[5,15,16]^ The height of the anterior and posterior sides is closely related to the formation of different vertical bone surface shapes, and the condyle, as the center of mandibular growth and development, plays a nonnegligible role in the formation of posterior height, so we speculate that the height of the condyle may not be the main reason for the difference in vertical growth patterns in class II patients. The comparison results of the long-axis angle of the condyle showed that the high-angle group was the smallest, and the correlation analysis showed that the long-axis angle of the condyle was negatively correlated with MP-FH, which also indicated that the joint stability of the high-angle patients was poor compared with the other groups.

In the comparison of the fossa morphology of each group, the depth and AEI were the largest in the low-angle group and the smallest in the high-angle group. Correlation analysis showed that MP-FH was negatively correlated with FD and inclination. The results of the study showed that the skeletal class II low-angle group had a deep and steep fossa morphology, while the high-angle group had a shallow and gentle fossa. Other studies have also observed differences in FD and AEI groups in different bony types, with low-angle patients having high, steep fossa and high-angle patients having low, flat fossa.^[16–18]^ Scholars believe that this mode of development has an important impact on the bony facial type, and the low flatness of the articular fossa weakens the limiting effect on the condyle, making it easier for the condyle to rotate in a clockwise direction, thus forming a high-angle bony facial shape. Conversely, a tall, steep temporomandibular fossa causes the jaw to rotate in the opposite direction, resulting in a low-angle facial morphology.^[17]^ In this study, no significant difference was found in the width of the fossa between the groups, which was consistent with the results of most previous studies.^[17,19]^

The position of the condyle-fossa has always been a hot topic in TMJ research, but the comfortable position of the condyle in the fossa is still controversial. Looking back at the previous literature, the prevailing view on the position of the condyle in the fossa is that the condyle without TMD with normal occlusion is predominantly in the median position.^[20]^ We used CBCT combined with Invivo 5.3 software to measure the joint space, and the results showed that the anterior and posterior AS in the control group were similar, and the condyle was basically in the median position. In addition, we compared the SS of each group and found that the low-angle group was the largest and the high-angle group was the smallest, which to a certain extent indicated that the vertical position of the condyle in the articular fossa was affected by the vertical bony surface type, and the condylar position was relatively high in patients with vertical growth and relatively low in patients with low angle. Correlation analysis showed that MP-FH was negatively correlated with SS, which was consistent with the differences between the above groups. The research of foreign scholars^[18]^ also supports our conclusions. This may be related to the strength of the masticatory muscles in patients with low angles, and the enhancement of masseter muscle function leads to the aggravation of counterclockwise rotation of the mandible, resulting in a smaller mandibular angle and stretching the condyle to a low position, forming a large SS and promoting condylar development. On the other hand, high-angle patients have a narrow SS, which may mean that the posterior area of the disc is thinned, thereby weakening the binding force on the disc, which may increase the risk of disc displacement, leading to structural and functional disorders of TMJ, resulting in TMD, which is also consistent with the findings that patients with bony class II high-angle patients are at high risk of TMD.^[21]^ Therefore, when designing orthodontic treatment plans in clinical practice, for high-angle patients, we need to increase the posterior height to increase the SS, avoid further reducing the upper space, and try to adjust the position of the condyle to the normal position to avoid causing TMJ lesions. Compared with the prearticular space, the results showed that the high-angle group was the largest, the low-angle group was second, the average-angle group was in the middle position, and the control group was the smallest, but the difference between the average-angle group and the control group was not statistically significant, while the comparison results of the retroarticular space showed that the low-angle group was the largest, the control group was the second, the average angle group was in the middle position, and the high-angle group was the smallest. In the distribution of condyle positions, the condyles in the control group were mainly in the median and anterior positions, the median and posterior positions in the low-angle group, the posterior position in the high-angle group, and the anterior and posterior positions in the average angle group. Correlation analysis showed that ANB angle was positively correlated with anterior AS, MP-FH was negatively correlated with PS, and MP-FH was significantly positively correlated with anterior AS. This indicates that patients with bony class II tend to have a relatively posterior condylar position, and compared with other groups, the high-angle group had a posterior displacement of the condyle, while the low-angle group had a larger joint space. Zhou and Liu^[22]^ and Chae et al^[12]^ came to the same conclusion as we did, that the condyle is positioned further posteriorly when the jaw rotates clockwise. Other studies have shown^[23]^ that patients with bony class II malocclusion usually have a steeper occlusal plane angle if the mandibular plane angle is larger, and the steeper the occlusal plane angle, the greater the anterior AS, and the posterior condyle in the sagittal position of the fossa, which also confirms our findings. In patients with low angles, the growth and development of the condyle is mainly upward and forward, which will cause the condyle to move backward to a certain extent. In addition, we found that although the control group and the bony class II homogeneous group had the same vertical bony surface shape, the condyle position in the control group was more distributed in the central and anterior positions, while the condyle position in the bony class II homogeneous group was more predominant in the anterior and posterior positions, which showed that the anteroposterior position of the condyle in the articular fossa was affected by the sagittal bone-to-bone surface shape.

In summary, there are certain differences in the morphology and location of different vertical bony TMJ in adult women with bone class II.

## 5. Conclusion

The morphology and position of bilateral TMJ were basically symmetrical between the skeletal class II adult female and the control group, and the position and morphology of the TMJ in the vertical skeletal type of adult female with bony class II were different.

## Author contributions

**Funding acquisition:** Xin Li.

**Writing—review & editing:** Xin Li.

**Conceptualization:** Ke Zhang.

**Formal analysis:** Ke Zhang.

**Methodology:** Ke Zhang.

**Software:** Ke Zhang.

**Writing—original draft:** Ke Zhang.

**Data curation:** Guannan Wang.

**Resources:** Haojie Hu.

**Validation:** Zhiqian Li.

**Visualization:** Zhiqian Li.
